# Online videos of robotic-assisted cholecystectomies: more harm than good?

**DOI:** 10.1007/s00464-024-11054-9

**Published:** 2024-07-15

**Authors:** Riley Brian, Camilla Gomes, Adnan Alseidi, Irving Jorge, Cris Malino, Eric Knauer, Domenech Asbun, Shanley B. Deal, Ian Soriano

**Affiliations:** 1 Department of Surgery, University of California San Francisco, 513 Parnassus Ave, S-321, San Francisco, CA 94143 USA; 2 Department of Surgery, Mayo Clinic, Phoenix, AZ USA; 3 Rural Physicians Group, Greenwich Village, CO USA; 4 Department of Surgery, Emory University School of Medicine, Atlanta, GA USA; 5 Hepatobiliary & Pancreatic Surgery, Miami Cancer Institute, Miami, FL USA; 6 Department of Surgery, Virginia Mason Medical Center, Seattle, WA USA

**Keywords:** Robotic surgery, Cholecystectomy, Online education, Video-based education

## Abstract

**Background:**

Many surgeons use online videos to learn. However, these videos vary in content, quality, and educational value. In the setting of recent work questioning the safety of robotic-assisted cholecystectomies, we aimed (1) to identify highly watched online videos of robotic-assisted cholecystectomies, (2) to determine whether these videos demonstrate suboptimal techniques, and (3) to compare videos based on platform.

**Methods:**

Two authors searched YouTube and a members-only Facebook group to identify highly watched videos of robotic-assisted cholecystectomies. Three members of the Society of American Gastrointestinal and Endoscopic Surgeons Safe Cholecystectomy Task Force then reviewed videos in random order. These three members rated each video using Sanford and Strasberg’s six-point criteria for critical view of safety (CVS) scoring and the Parkland grading scale for cholecystitis. We performed regression to determine any association between Parkland grade and CVS score. We also compared scores between the YouTube and Facebook videos using a *t* test.

**Results:**

We identified 50 videos of robotic-assisted cholecystectomies, including 25 from YouTube and 25 from Facebook. Of the 50 videos, six demonstrated a top-down approach. The remaining 44 videos received a mean of 2.4 of 6 points for the CVS score (SD = 1.8). Overall, 4 of the 50 videos (8%) received a passing CVS score of 5 or 6. Videos received a mean of 2.4 of 5 points for the Parkland grade (SD = 0.9). Videos on YouTube had lower CVS scores than videos on Facebook (1.9 vs. 2.8, respectively), though this difference was not significant (*p* = 0.09). By regression, there was no association between Parkland grade and CVS score (*p* = 0.13).

**Conclusion:**

Publicly available and closed-group online videos of robotic-assisted cholecystectomy demonstrated inadequate dissection and may be of limited educational value. Future work should center on introducing measures to identify and feature videos with high-quality techniques most useful to surgeons.

**Graphical abstract:**

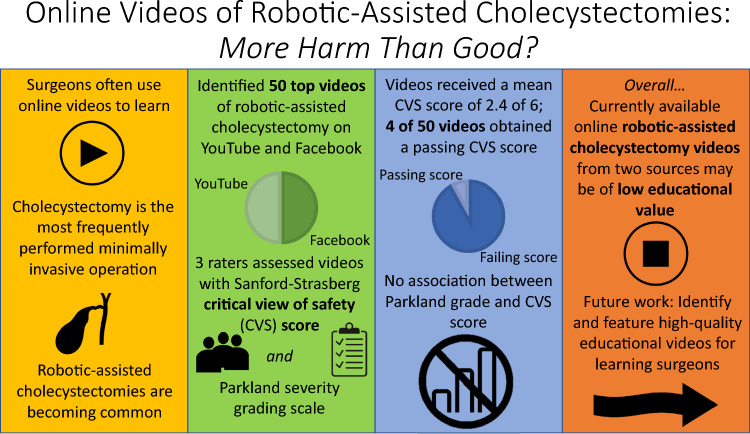

Many surgeons use publicly available online videos to learn and review prior to operations [1]. Unfortunately, the substantial variation of operative techniques shown in such videos challenges this practice [2, 3]. A general lack of peer review, with concomitant misleading or incomplete information, has featured prominently in recent media reports [4, 5]. As minimally invasive operations can be easily recorded and shared, videos portraying low-quality techniques in minimally invasive surgery may be particularly susceptible to dissemination [6–9].

Minimally invasive cholecystectomy claims a central role in surgical training. Considered a core procedure by the American Board of Surgery, cholecystectomy far exceeds all other operations as the most frequently performed minimally invasive operation by general surgery trainees [10, 11]. Though the majority of cholecystectomies are still performed laparoscopically, robotic-assisted cholecystectomy has emerged as the most common robotic procedure in general surgery [12]. Some have advocated for harnessing this procedure to allow trainees and practicing surgeons new to robotic surgery to gain experience [13, 14].

While multiple authors have published operative steps for robotic-assisted cholecystectomy, it is unknown how most learners are being taught to perform the procedure [14–16]. Indeed, credentialing and training processes for robotic surgery vary widely across different institutions, with no accepted, standardized pathway [17]. Cumulative sum analysis has suggested a learning curve of up to 134 patients in robotic-assisted cholecystectomy though some authors have noted a more “minimal” learning curve [18–20].

A recent retrospective study, though limited by potential confounders, found that the incidence of bile duct injury needing operative repair was higher in robotic-assisted (0.7%) than in laparoscopic (0.2%) cholecystectomy [21]. This finding raises questions about some surgeons’ view of robotic-assisted cholecystectomies as ‘learning cases’ that are safer than laparoscopic cholecystectomies [15, 22, 23]. Notably, however, a systematic review of robotic-assisted versus laparoscopic cholecystectomies identified challenges associated with comparing complications when including cases performed during surgeons’ initial learning [24].

Nonetheless, this recent finding of higher bile duct injury in robotic-assisted compared to laparoscopic cholecystectomies, coupled with renewed scrutiny over online learning from surgical videos, calls into question the quality of techniques in online videos used by surgeons who are learning robotic-assisted cholecystectomy. As such, we aimed (1) to identify highly watched online videos of robotic-assisted cholecystectomies, (2) to determine whether these videos demonstrate suboptimal techniques, and (3) to compare videos based on platform.

## Methods

### Video selection

Two authors (RB, CG) searched YouTube and the Robotic Surgery Collaboration on Facebook, a members-only robotic surgeon Facebook group, in February 2024 to identify robotic-assisted cholecystectomies. We used combinations of search terms including “robotic-assisted,” “robotic,” “intra-operative,” “gallbladder,” “cholecystectomy,” “chole,” “da Vinci,” and “daVinci” with the aim of including the YouTube videos with the most views and the Facebook videos with the most reactions (i.e., the sum of “like,” “love,” “haha,” “wow,” “sad,” and “angry”). If a Facebook post linked to YouTube, we included this as a YouTube video. We chose to focus on these videos to allow us to evaluate the most widely used material. After independently identifying videos, the two researchers reconciled their lists of videos with the most views and reactions.

We included videos that involved robotic-assisted cholecystectomy and showed intra-operative views of the hepatocystic triangle dissection with the clipping or ligation of the cystic duct and artery. We excluded videos of remnant cholecystectomies and takedowns of cholecystoenteric fistulas. We reviewed videos with associated procedures, such as bile duct exploration or cholangiogram, and videos that had been edited, so long as they showed the intra-operative views of a cholecystectomy as above. We included videos regardless of language or country of origin.

After confirming the list of included videos, we created segments of each video that spanned from the final dissection of the hepatocystic triangle to the placement of the first clip or suture. Other than de-identifying videos by removing out-of-body shots, we did not edit the video content or speed during this period, allowing for the same experience of the videos’ visualization of the critical view of safety (CVS) as other online video watchers.

### Video review

We graded the videos using Sanford and Strasberg’s six-point criteria for CVS scoring [25]. A score of five or six within this scoring system is considered passing and safe. We chose to use Sanford and Strasberg’s scoring criteria given their widespread use in previously published work and the ease of using the system. As with previously published work assessing cholecystectomy videos, we awarded points if the videos showed components of the CVS in either the anterior or the posterior view since most videos did not include a posterior view [26]. We also evaluated each case using the Parkland grading scale for cholecystitis as a potential marker of case difficulty [27]. We chose to use the Parkland grading scale as it is a simple and operative-based system, though grading may have been limited by the available video footage [28]. Furthermore, we reviewed the comments posted by each video author to determine whether the poster included a question or a request for suggestions, tips, or feedback from viewers. This allowed us to better contextualize the purpose of the videos.

After reviewing the scoring criteria and going through rater training using four published practice cases [25], three members of the Society of American Gastrointestinal and Endoscopic Surgeons (SAGES) Safe Cholecystectomy Task Force who were also members of the Robotic Surgery Collaboration Facebook group independently reviewed all videos in random order. We averaged the scores and grades assigned by the three video reviewers for description and analysis.

### Statistical analysis

Based on previously published work assessing laparoscopic cholecystectomy videos, we determined that including 50 videos would provide more than 90% power to detect a one-point CVS score difference between YouTube and Facebook videos [26]. We generated descriptive data about the included videos and the assigned scores. We calculated inter-rater reliability with Krippendorff’s alpha [29]. We performed linear regression to determine any association between Parkland grade and CVS score. We compared scores between the YouTube and Facebook videos using a t test after confirming that the data were normally distributed using a Shapiro–Wilk test. We set statistical significance at *p* < 0.05. We performed all analyses in Stata/IC 16.1 for Mac (StataCorp, College Station, TX).

### Ethical approval

The University of California San Francisco Institutional Review Board exempted this study from review (IRB23-40322). Group leadership from the Robotic Surgery Collaboration on Facebook approved this study.

## Results

We identified 50 online videos of robotic-assisted cholecystectomies, including 25 from YouTube and 25 from Facebook. Videos represented work by 36 unique surgeons. Of the 50 videos, 48 videos showed operations with da Vinci robotic surgical systems (Intuitive Surgical, Sunnyvale, CA) while two videos showed operations using other systems. YouTube videos had a mean of 24,832 views (SD = 69,236; range = 3067–352,312). Facebook videos had a mean of 74 reactions (SD = 26; range = 47–146). Through review of comments posted with videos, we found that one of the 50 videos included a question from the surgeon for viewers. Of the remaining 49 videos, none contained a question or request for suggestions, tips, or feedback.

**Fig. 1:**
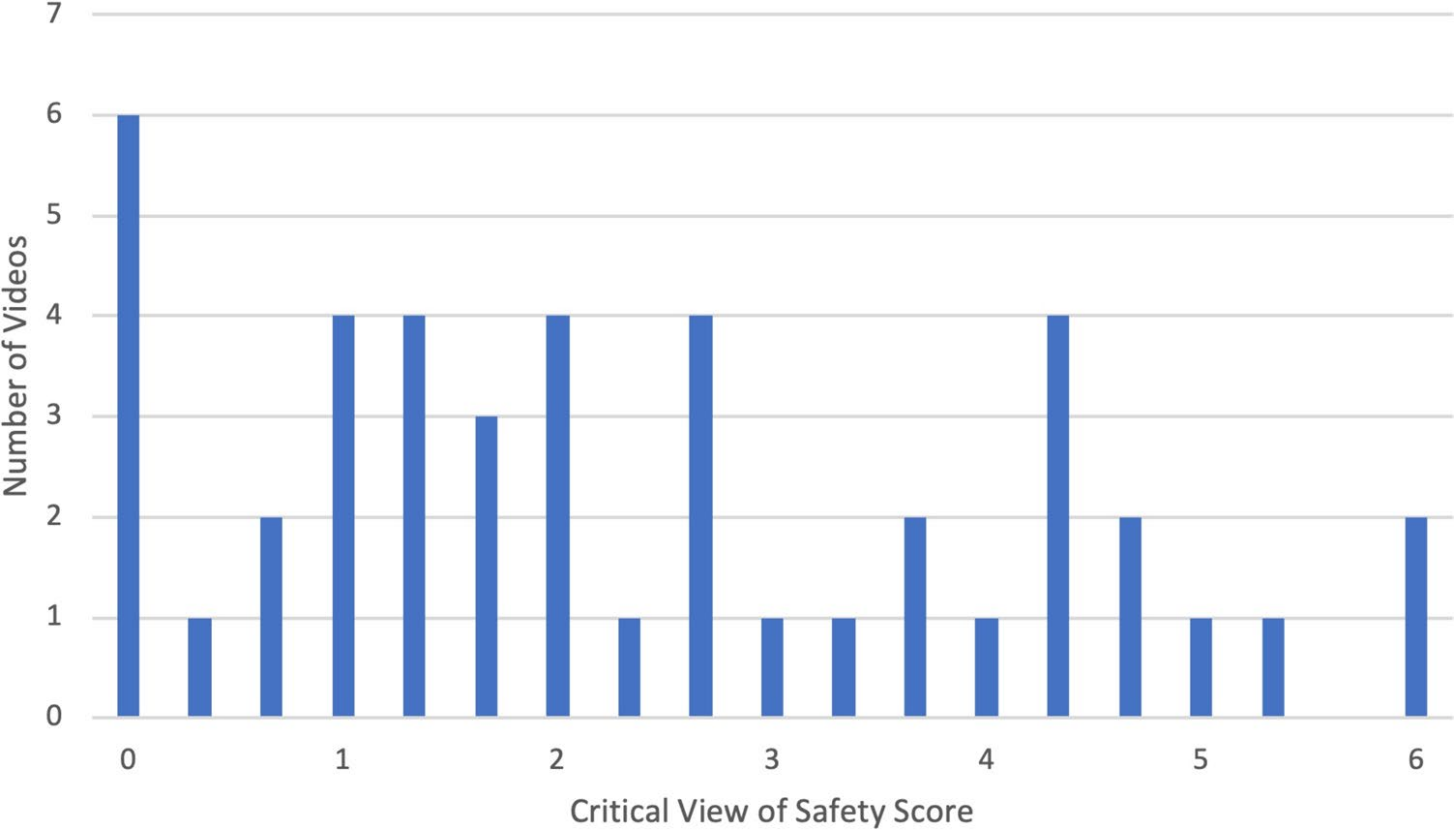
Critical View of Safety (CVS) scores of the videos taking a traditional (infundibulum-first) approach to dissection. Videos taking a top-down (fundus-first) approach were not included here. Scores were averaged based on three raters’ video review

**Fig. 2:**
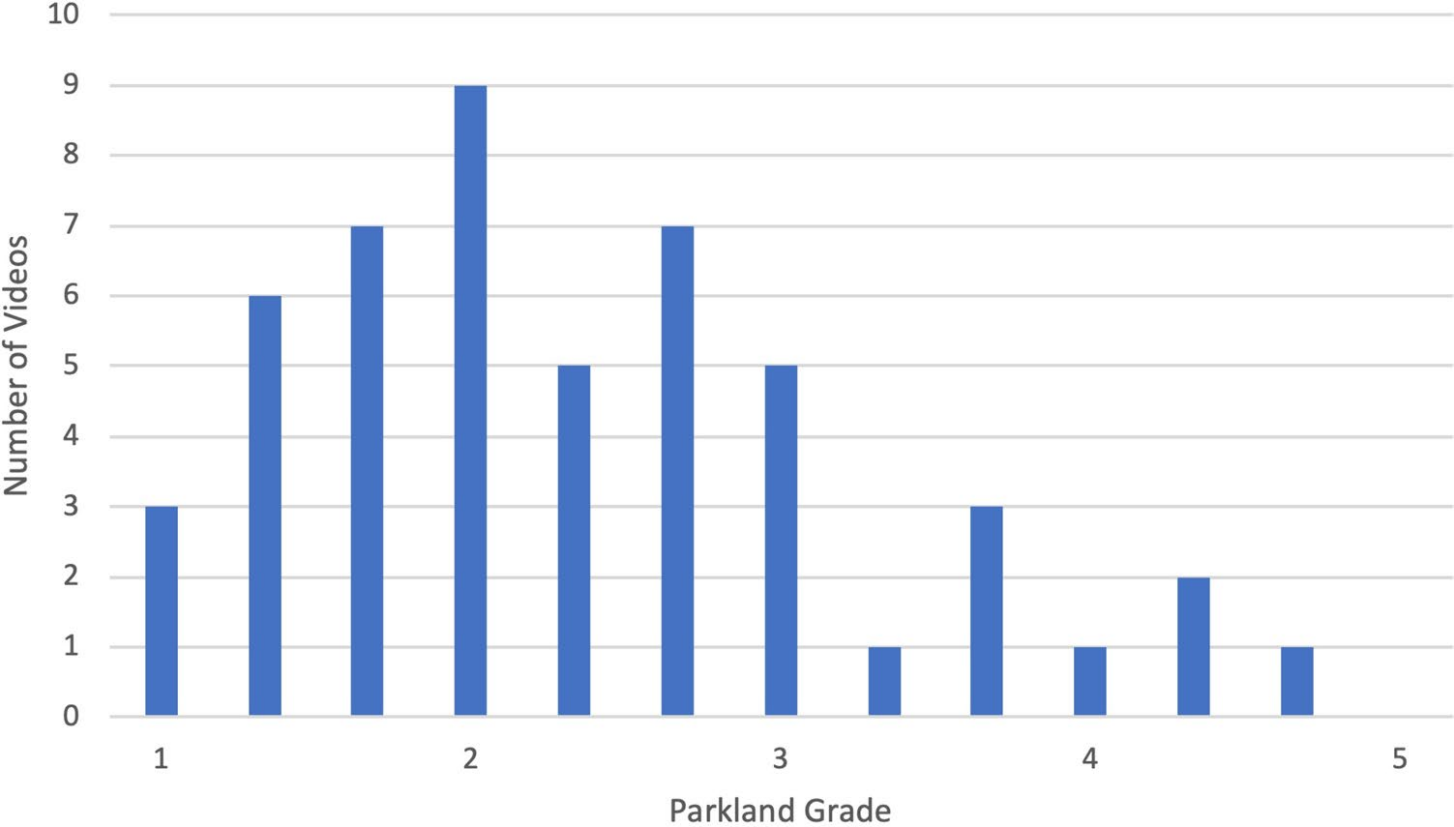
Parkland grades of all videos. Grades were averaged based on three raters’ video review

**Fig. 3:**
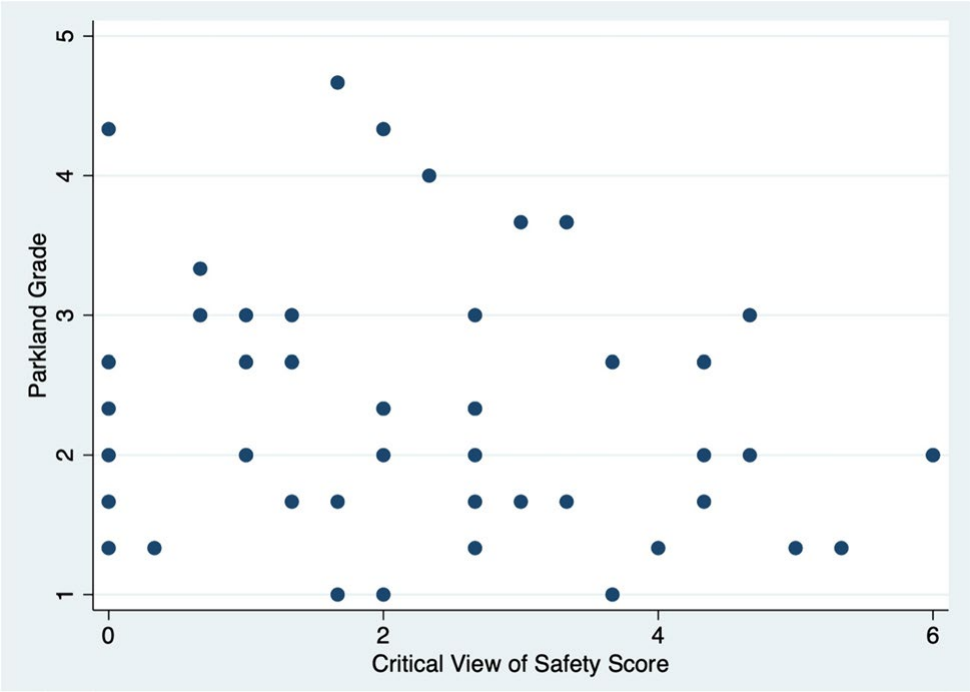
Parkland grade was not associated with Critical View of Safety (CVS) score

Three raters assigned CVS scores and Parkland grades to the 50 videos, with an inter-rater reliability of 0.44 for CVS scores and 0.49 for Parkland grades. Six of the videos demonstrated a top-down (or fundus-first) approach. The remaining 44 videos, which took a traditional (infundibulum-first) approach, received a mean CVS score of 2.4 of 6 points (SD = 1.8) (Fig. 1). Only 4 of the 50 videos (8%) received a passing CVS score of 5 or 6. The 50 videos received a mean of 2.4 of 5 points for the Parkland grade (SD = 0.9) (Fig. 2). By regression, there was no association between Parkland grade and CVS score (*p* = 0.13) (Fig. 3).

**Fig. 4:**
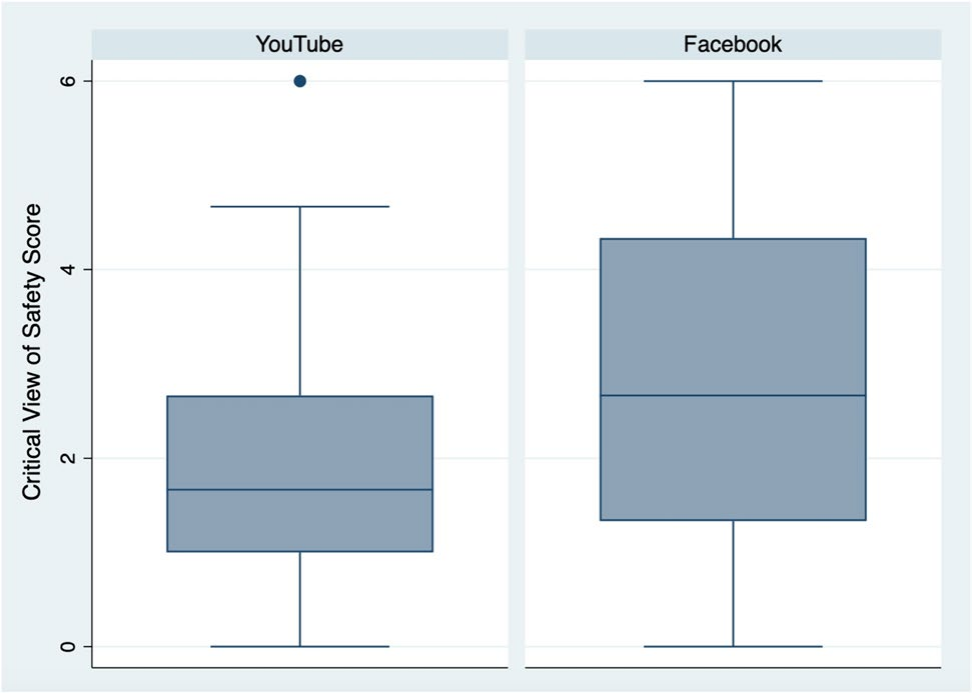
Critical View of Safety (CVS) scores of the rated YouTube and Facebook videos. Scores were not significantly different (*p* = 0.09)

Videos on YouTube had lower CVS scores than videos on Facebook (1.9 vs. 2.8, respectively), though this difference was not statistically significant (*p* = 0.09) (Fig. 4). YouTube and Facebook videos had similar Parkland grades (2.1 vs. 2.3, respectively, with *p* = 0.52).

## Discussion

In this study, we identified that the vast majority of the highly viewed online videos of robotic-assisted cholecystectomy—both publicly available and from a closed group—did not attain passing CVS scores and demonstrated suboptimal techniques. Furthermore, we found that the grade of cholecystitis did not correlate with the CVS score, as several videos received low CVS scores even in the setting of normal anatomy and no inflammation. These findings together suggest that most available online videos of robotic-assisted cholecystectomy are inadequate as educational material.

This study adds to significant prior work that has evaluated the content, value, and accuracy of online surgical videos. Most prior studies have collated videos from a single online source, with YouTube being the most commonly used site. Previous authors have reviewed laparoscopic cholecystectomies and have identified very low educational quality and CVS scores among included videos. Interestingly, the scores of the robotic-assisted cholecystectomy videos that we reviewed were similar to those of laparoscopic cholecystectomy videos reviewed in two prior studies [26, 30]. Given the low CVS scores for both laparoscopic and robotic-assisted cholecystectomy, it remains unclear that learning from online videos contributes to differential performance in these two procedures. Of note, the rating process did differ between these studies, which limits direct comparisons. Other authors have reviewed robotic-assisted procedures and have found substantial variation in the included videos [31, 32].

Video review can be an extremely effective educational tool for learning robotic-assisted surgery [33]. Based on the theory of multimodality, multiple modes—including visual, audio, and written—may facilitate learning better than a single mode alone [34, 35]. Using personal and others’ videos has shown promise in preparing surgical learners for the operating room [36, 37]. Prior authors have described how best to design videos to maximize their educational impact [38]. However, as we and others have demonstrated, many existing case review videos demonstrate suboptimal practices. Unfortunately, this problem appears to persist across video sources. We found videos with inadequate dissection on two platforms and another prior study showed that curated videos, including from a society-associated video repository, contained lower quality videos than YouTube [39]. Societies should carefully review videos’ content and consider the above education-focused recommendations to use their platforms to promote high-value videos.

Several limitations moderate the interpretation of the findings in this study. We included a limited number of videos from two platforms, and videos from Facebook came from one group. A larger video corpus could allow us to detect smaller differences in scores among video platforms. Surgeons and trainees watch and upload numerous online videos in other platforms, and they may do so for many reasons other than viewer education—such as obtaining feedback on operative technique or practicing with video editing. While most video posters did not include a question or a request for suggestions, tips, or feedback, we do not know surgeons’ unstated intentions in posting their videos. Furthermore, we used only one rating system to score videos. While prior authors have similarly used this rating system, there are numerous other ways to evaluate videos. Additionally, the inter-rater reliability was moderate for both CVS score and Parkland grade. We took the average of the three raters’ scores and grades for our analyses. Lastly, raters did not have the three-dimensional view of an operating robotic surgeon, and thus the CVS scores assigned may not reflect the actual views obtained intra-operatively. However, we aimed to evaluate videos’ educational value to online viewers—who also lack a three-dimensional view.

Overall, we found that the included online videos of robotic-assisted cholecystectomy demonstrated inadequate dissection and may be of limited educational value. Future work should center on introducing measures to identify and feature high-quality videos most useful to surgeons.
